# Organizational citizenship behavior of clinical nurses: A systematic review and meta-analysis

**DOI:** 10.1097/MD.0000000000041755

**Published:** 2025-03-14

**Authors:** Jia-Xi Sun, Fu-Yan Liu, Wen-Nv Hao

**Affiliations:** a School of Nursing, Inner Mongolia Medical University, Hohhot, Inner Mongolia, China; b Department of Emergency, Affiliated Hospital of Inner Mongolia Medical University, Hohhot, Inner Mongolia, China.

**Keywords:** clinical nurses, meta-analysis, organizational citizenship behavior, systematic review

## Abstract

**Background:**

Organizational citizenship behavior can improve work efficiency and employee participation. This study systematically evaluated the level of organizational citizenship behavior of clinical nurses and meta-analysis the factors that affect it in terms of personal characteristics. The conclusions provide valuable recommendations for nursing managers to focus on cultivating organizational citizenship behavior.

**Methods:**

Adhering to the preferred reporting items for Systematic Reviews and Meta-analyses guidelines, we searched PubMed, Cochrane Library, Web of Science, Embase, ScienceDirect, Wiley Online Library, Scopus, China National Knowledge Infrastructure, SinoMed, Wanfang and VIP 11 databases from the inception of the databases until November 2023. Subsequently, 2 researchers independently screened the literature, extracted data, and evaluated the risk of bias in the included studies. Meta-analysis was performed using Stata 15.0 and RevMan 5.4 software.

**Results:**

Twenty cross-sectional studies were included. The sample comprised 8657 nurses. The results of the meta-analysis showed that marital status, employment types, participation in mental health-related training, professional titles, and years of work experience affected nurses’ organizational citizenship behavior were the influencing factors of nurses’ organizational citizenship behavior (*P* < .05).

**Conclusion:**

The findings suggest that the level of organizational citizenship behavior among nurses is relatively high, but it still needs to be maintained. This result suggests that nursing managers should pay more attention to cultivating nurses’ organizational citizenship behavior to improve organizational efficiency and further improve the quality of high-quality nursing services.

## 1. Introduction

With the transformation of the medical model and the deepening of high-quality nursing services, the National Health Commission has emphasized that medical institutions should mobilize the work enthusiasm of nursing staff.^[1]^ However, there are problems in the current medical environment, such as high work pressure, lack of nursing talent resources, and a high risk of occupational injuries.^[2]^ These problems lead most nurses only to complete the work within their responsibilities and neglect to take the initiative to show behaviors that benefit other colleagues in the organization or team.^[3]^ Therefore, arousing employees’ enthusiasm for work has become a problem that managers need to solve.

Organizational citizenship behavior (OCB) is one of the important forms of work enthusiasm.^[4]^ OCB refers to voluntary behavior an employee displays that is not part of the formal task requirements and duties, which are not stated in the job description, and they benefit others and the organization.^[4]^ Although the organizational compensation system does not directly recognize it, it maintains and enhances the psychosocial environment in which tasks are performed.^[5]^

OCB has a positive effect on improving employee loyalty, reducing turnover rates, and maintaining team stability.^[6]^ As an important driving factor in organizational development and the deepening of high-quality nursing services, OCB can effectively coordinate teamwork and improve organizational performance.^[7]^ Therefore, exploring nurses’ organizational citizenship behavior has significant practical implications.

At present, there is a lack of comprehensive research on the level and influencing factors of organizational citizenship behavior of clinical nurses. Therefore, this study is the first to use systematic review and meta-analysis to evaluate the research on nurses’ organizational citizenship behavior comprehensively. The conclusions provide valuable recommendations for nursing managers to improve organizational efficiency and better quality nursing services.

## 2. Materials and methods

### 2.1. Design and registration

This review was conducted in accordance with the Preferred Reporting Items for Systematic Reviews and Meta-Analyses (PRISMA 2020) recommendations.^[8]^ The protocol was registered with PROSPERO (registration number: CRD42024580001).

### 2.2. Information sources

**Figure 1.**
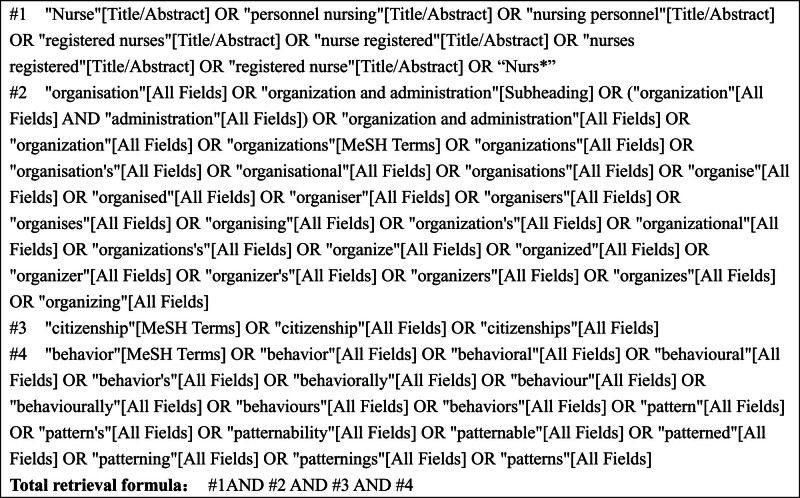
Search strategy.

A preliminary search was undertaken in the Cochrane Library and PROSPERO website to confirm no systematic reviews or meta-analysis address the level and influencing factors of organizational citizenship behavior among nurses. Then, 2 reviewers formulated the search strategy. All retrieval methods are based on a combination of medical subject headings (MeSH terms) and free words, and are adjusted according to a specific database. The search strategy included combinations of 4 key terms (nurses; registered nurses; clinical nurses; organizational citizenship behavior). The literature on the organizational citizenship behavior of nurses was searched from the inception to November 30, 2023 in 11 databases: PubMed, Web of Science, EMBASE, Cochrane Library, ScienceDirect, Wiley Online Library, Scopus, China National Knowledge Infrastructure, Chinese Biological Medical (CBM), WanFang and Weipu Database. Using PubMed as an example, the specific search strategy is illustrated in Figure 1.

### 2.3. Eligibility criteria

The inclusion criteria were as follows: study design: a cross-sectional study; participants: clinical nurses; instrument: used the organizational citizenship behavior related assessment scale as an instrument for measuring organizational citizenship behavior levels; outcomes: reported the score and influencing factors of organizational citizenship behavior; language: were written in English or Chinese; others: had mixed samples from healthcare, whereas clearly reported independent data for nursing staff.

The exclusion criteria were as follows: design: case reports, conference abstracts, reviews, protocols, commentaries, and letters to the editor; participants: auxiliary nurses, nursing students, nursing educators or other equivalents; outcomes: scores for OCB were not provided; others: full text not available and low-quality articles.

### 2.4. Study selection and data extraction

Two researchers (SJX and LFY) independently screened the literature, extracted data, and cross-checked the data. Any objections should be discussed or resolved through negotiations with a third party (HWN). When selecting the articles, we first read the title and abstract. After excluding irrelevant articles, we read the full text to determine whether they were included. Data extraction included basic information about the included studies: first author, publication year, sample size, source region, etc; outcome indicators: reported the score and influencing factors of organizational citizenship behavior; and the related elements of bias risk assessment.

### 2.5. Quality appraisal

Two reviewers(SJX and LFY), in the form of mutual blindness, independently evaluated the included literature using the American Agency for Health Care Research and Quality tool.^[9]^ The Agency for Health Care Research and Quality tool mainly consists of 11 items (study design, participants, variables, data, bias, etc), which were imported into Revman5.4 software to evaluate the quality and bias risk of the literature. Red in figure indicates “No” (high risk), yellow represents unclear risk and green means “Yes” (low risk). If the answer is “No” (high risk) or “Unclear,” the item score is “0”; if the answer is “Yes” (low risk), the item score is “1.” A score of 8 to 11 was considered high quality, 4 to 7 as moderate quality, and <4 as low quality. After independent evaluation, 2 researchers (SJX and LFY) discussed and reached a consensus. If there is any disagreement, the third researcher (HWN) will arbitrate or the research group will discuss and decide.

### 2.6. Statistical analysis

Endnote 20 was used to summarize the articles. Excel software was used for data extraction, management, statistics, and descriptive analysis of the outcome indicators. Meta-analysis was performed using Stata 15.0 and RevMan 5.4 software. Continuous variables are represented by the standardized mean difference (SMD) and 95% confidence interval (95% CI). The Chi-square test and *I*² index were used to determine whether there was heterogeneity among studies. If there was no heterogeneity among the studies (*P* > .1, *I*² < 50%), a fixed-effect model was adopted. If there was heterogeneity among studies (*P* ≤ .1, *I*² ≥ 50%), the random-effects model was used to combine the effect sizes. Sensitivity analysis was performed by comparing the combined results of the 2 models to assess the stability of the results. Egger’s test was performed using Stata software (version 15) to assess the presence or absence of publication bias. *P* > .05 meant no publication bias.

### 2.7. Ethical consideration

Ethical approval was not required based on the use of previously published secondary data or the nature of the meta-analysis.

## 3. Results

### 3.1. Literature screening process and results

**Figure 2.**
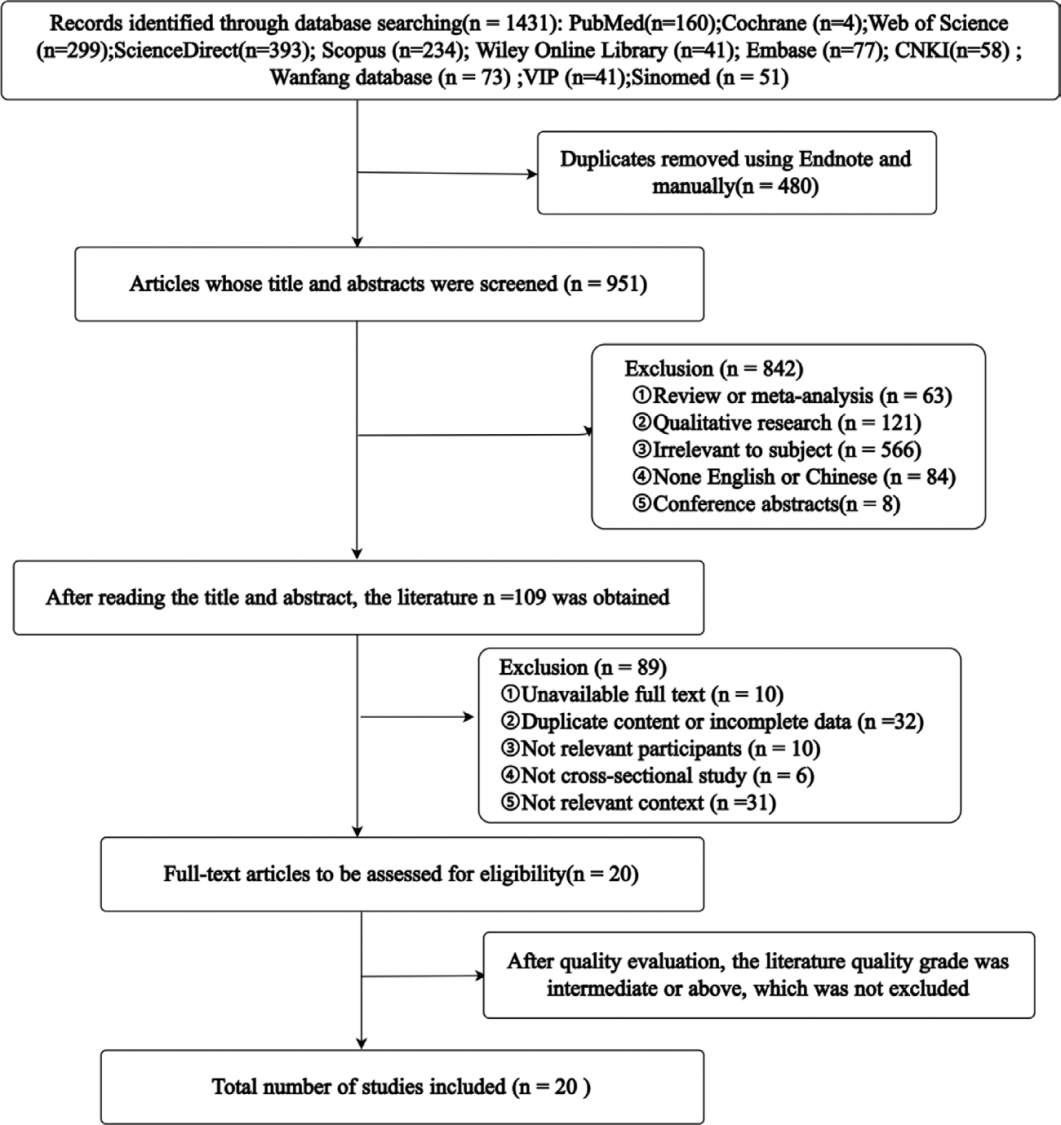
Literature screening process and results. CNKI = China National Knowledge Infrastructure, VIP = Weipu database.

A total of 1431 publications were searched from 11 electronic databases. After literature screening, 20 studies were included in this review.^[10–29]^ The PRISMA diagram of literature screening is presented in Figure 2.

### 3.2. Characteristics of included studies

The 20 included studies involved 8657 participants in total. Nineteen (95%) studies were conducted in Asia (China, Iran and Türkiye), and 1 (5%) was from South Africa. The basic characteristics of the included studies are presented in Table 1.

**Table 1:** Basic characteristics of included studies.

Author, year	Design	Region, country	Instrument	Sample size	Score, mean (SD)	Influencing factors
Leng et al 2019^[10]^	Cross-sectional study	Tianjing, China	*	206	101.19 ± 10.33	③⑪
Lin and Zhao et al 2016^[11]^	Cross-sectional study	Zhejiang, China	†	540	106.61 ± 25.74	⑫
Huang and Xie et al 2016^[12]^	Cross-sectional study	Jiangsu, China	†	436	94.04 ± 11.03	①③④⑤⑥⑦⑧⑨
Liu 2017^[13]^	Cross-sectional study	Henan, China	*	329	98.64 ± 7.44	④⑤
Jiang et al 2016^[14]^	Cross-sectional study	Heilongjiang, China	*	206	95.61 ± 14.70	③④⑤⑥⑨
Xiao 2020^[15]^	Cross-sectional study	Beijing, China	*	277	101.88 ± 16.36	③④⑤⑥⑧
Shi 2021^[16]^	Cross-sectional study	Jiangsu, China	*	179	100.64 ± 13.40	①③⑤
Ren 2015^[17]^	Cross-sectional study	Gansu, China	†	1001	85.00 ± 8.74	⑭
Yu et al 2019^[18]^	Cross-sectional study	Tianjing, China	‡	251	86.45 ± 14.21	①③④⑤⑥⑦
Xu and Cheng 2022^[19]^	Cross-sectional study	Shanghai, China	*	112	99.60 ± 10.08	⑯⑰
Lu et al 2020^[20]^	Cross-sectional study	Guangdong, China	*	565	103.76 ± 12.19	①②③④⑤⑥⑦⑧⑨
Du 2021^[21]^	Cross-sectional study	Jiangsu, China	*	384	97.44 ± 11.04	③⑤⑥⑦⑨⑫
Huang and Jiang 2012^[22]^	Cross-sectional study	Fujian, China	∥	200	86.65 ± 8.04	⑮
Sha 2023^[23]^	Cross-sectional study	Jiangsu, China	*	555	104.40 ± 11.28	①②③④⑤⑥⑦⑨
Aloustani et al 2020^[24]^	Cross-sectional study	Tehran, Iran	§	250	80.75 ± 16.22	⑪
Wang et al 2023^[25]^	Cross-sectional study	Shandong, China	†	1157	88.60 ± 8.80	④⑥⑦⑨⑪
Chamisa et al 2020^[26]^	Cross-sectional study	Eastern Cape, South Africa	§	228	113.76 ± 26.40	⑯
Jin et al 2022^[27]^	Cross-sectional study	Sichuan, China	*	606	101.57 ± 11.57	③④⑩⑯
Özlük B and Baykal 2020^[28]^	Cross-sectional study	Istanbul, Türkiye	§	429	130.80 ± 22.80	⑬⑭
Zeng et al 2023^[29]^	Cross-sectional study	Sichuan, China	*	746	101.47 ± 12.14	①③④⑥⑧⑩⑯

SD = standard deviation.

①Age, ②Gender, ③Marital status, ④Years of work experience, ⑤Professional title, ⑥Education level, ⑦Employment type, ⑧Monthly income, ⑨Position, ⑩Participation in mental health-related training, ⑪Ethical climate, ⑫Job embeddedness, ⑬Organizational trust, ⑭Job satisfaction, ⑮Organizational justice, ⑯Psychological capital, ⑰Job stressor.

Survey tools:

* Nurse Organizational Citizenship Behavior Scale (NOCBS) by Wan et al.

† Organizational Citizenship Behavior Scale (OCBS) by Farh et al.

‡ Medical Staff Organizational Citizenship Behavior Questionnaire by Bu et al.

§ Organizational Citizenship Behavior Questionnaire by Podsakoff et al.

∥ Organizational Citizenship Behavior Scale adapted by Ma et al.

### 3.3. Basic risk assessment results of included studies

**Figure 3.**
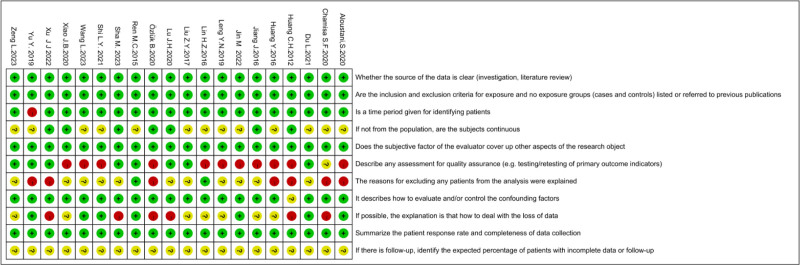
Risk of bias analysis of each included study.

**Figure 4.**
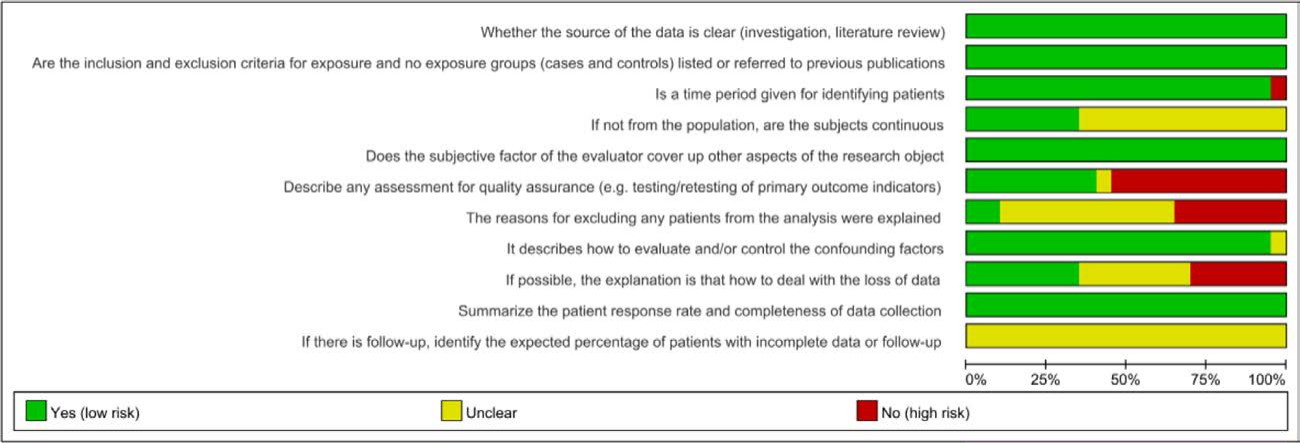
Overall risk of bias analysis of included studies.

Among these selected studies, 5 (25%) papers were evaluated as high quality (score of 8–11) and 15 (75%) papers were of moderate quality (score of 4–7). A detailed risk of bias assessment is shown in Figures 3 and 4.

### 3.4. Meta-analysis results

#### 3.4.1. Organizational citizenship behavior level

**Figure 5.**
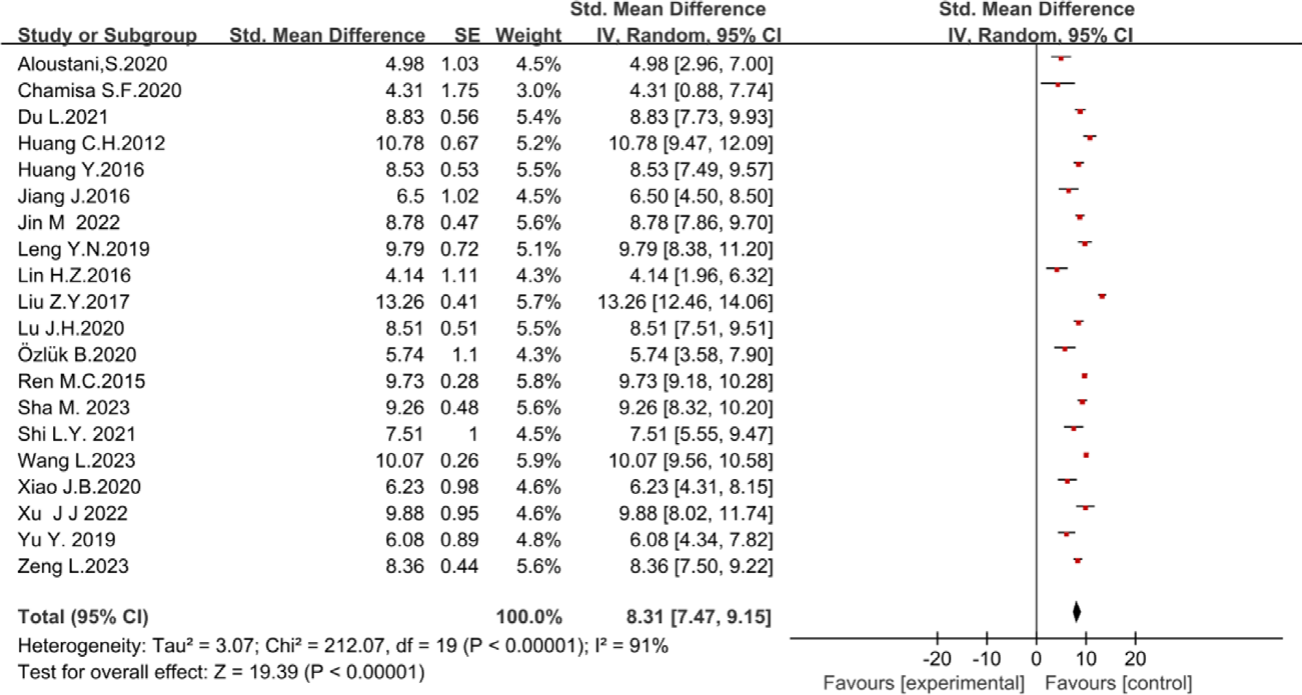
A meta-analysis of organizational citizenship behavior in nurses.

The overall level of organizational citizenship behavior among clinical nurses in this study was SMD = 8.31 (95%CI [7.47, 9.15]). Owing to the sizeable statistical heterogeneity (*P* < .00001, *I*² = 91%), a random effects model was used for the analysis, as shown in Figure 5.

#### 3.4.2. Influencing factors of organizational citizenship behavior

##### 3.4.2.1. Marital status

**Figure 6.**
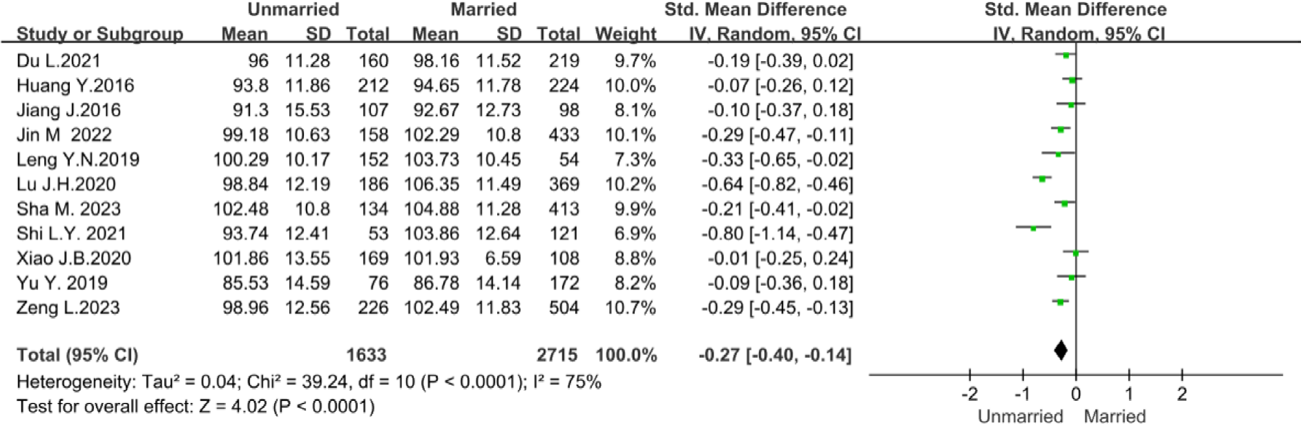
Forest plot of the influence of marital status on nurses’ organizational citizenship behavior.

To explore whether the marital status would impact the organizational citizenship behavior of nursing staff, a meta-analysis of the 11 included studies.^[10,12,14–16,18,20,21,23,27,29]^ Owing to the sizeable statistical heterogeneity (*P* < .0001, *I*² = 75%), a random effects model was used for the analysis. Meta-analysis showed that married nurses’ organizational citizenship behavior score was higher than unmarried nurses (*Z* = 4.02, *P* < .0001), as shown in Figure 6.

##### 3.4.2.2. Employment types

**Figure 7.**
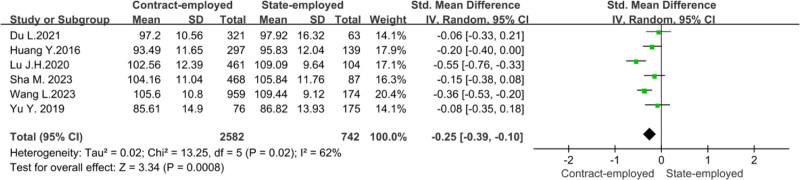
Forest plot of the influence of employment types on nurses’ organizational citizenship behavior.

To explore whether the employment types would impact the organizational citizenship behavior of nursing staff, researchers conducted a meta-analysis in 6 studies.^[12,18,20,21,23,25]^ Owing to the sizeable statistical heterogeneity (*P* = .02, *I*² = 62%), a random effects model was used for the analysis. Meta-analysis showed that the score of organizational citizenship behavior of state-employed nurses was higher than that of contract-employed nurses (*Z* = 3.34, *P* = .0008), as shown in Figure 7.

##### 3.4.2.3. Educational level

**Figure 8.**
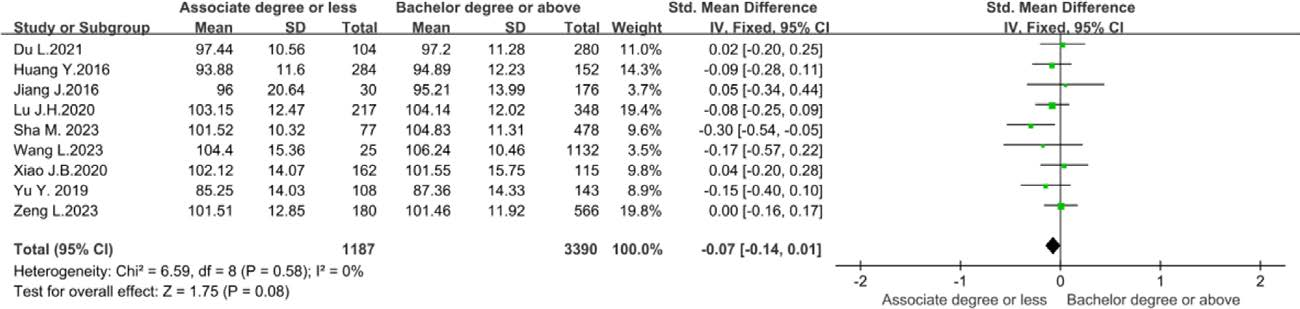
Forest plot of the effect of educational level on nurses’ organizational citizenship behavior.

To explore whether the educational level would impact the organizational citizenship behavior of nursing staff, researchers conducted a meta-analysis in 9 studies.^[12,14,15,18,20,21,23,25,29]^ As there was no statistical heterogeneity (*P* = .58, *I*² = 0%), a fixed effects model was used for the analysis. The results of the meta-analysis showed no statistically significant difference in the scores of organizational citizenship behavior of nurses with different educational backgrounds (*Z* = 1.75, *P* = .08), as shown in Figure 8.

##### 3.4.2.4. Participation in mental health-related training

**Figure 9.**
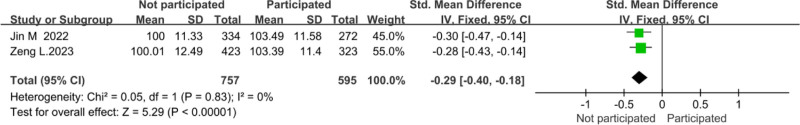
Forest plot of the effect of participation in mental health-related training on nurses’ organizational citizenship behavior.

To explore whether participation in mental health-related training would impact the organizational citizenship behavior of nursing staff. Researchers conducted a meta-analysis in 2 studies.^[27,29]^ As there was no statistical heterogeneity (*P* = .83, *I*² = 0%), a fixed effects model was used for the analysis. Meta-analysis showed that nurses who participated in mental health-related training had a higher score of organizational citizenship behavior, and the difference was statistically significant (*Z* = 5.29, *P* < .00001), as shown in Figure 9.

##### 3.4.2.5. Professional titles

**Figure 10.**
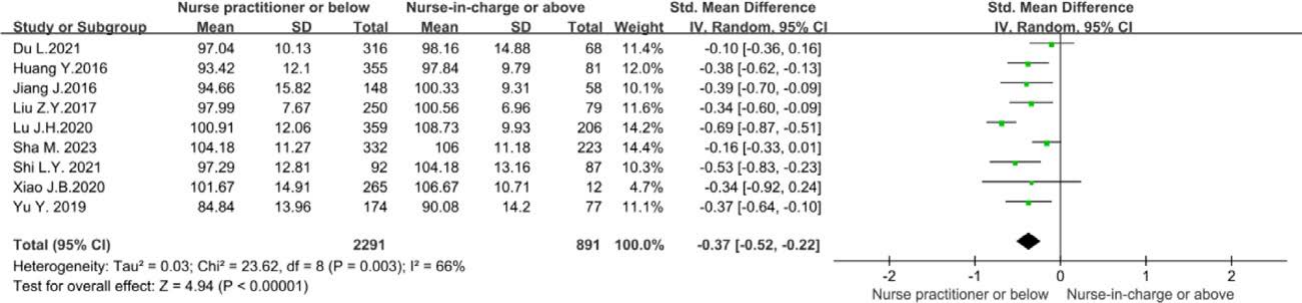
Forest plot of the influence of professional titles on nurses’ organizational citizenship behavior.

To explore whether the professional titles would impact the organizational citizenship behavior of nursing stuff. Researchers conducted a meta-analysis in 9 studies.^[12–16,18,20,21,23]^ Owing to the sizeable statistical heterogeneity (*P* = .003, *I*² = 66%), a random effects model was used for the analysis. Meta-analysis showed that the score of organizational citizenship behavior of nurses with the professional title of supervisor or above was higher (*Z* = 4.94, *P* < .00001), as shown in Figure 10.

##### 3.4.2.6. Years of work experience

**Figure 11.**
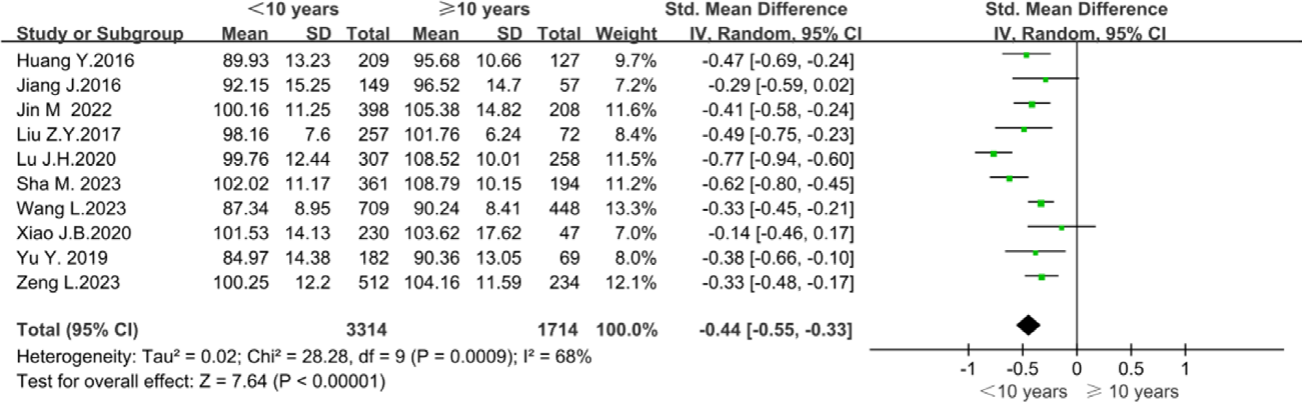
Forest plot of the effect of years of work experience on nurses’ organizational citizenship behavior.

To explore whether years of work experience would impact the organizational citizenship behavior of nursing staff. Researchers conducted a meta-analysis in 10 studies.^[12–15,18,20,23,25,27,29]^ Owing to the large statistical heterogeneity (*P* = .0009, *I*² = 68%), a random effects model was used for the analysis. Meta-analysis showed that nurses with ≥10 years of work experience had higher scores of organizational citizenship behavior (*Z* = 7.64, *P* < .00001), as shown in Figure 11.

### 3.5. Sensitivity analysis

Sensitivity analysis was performed to assess the robustness of the results. Sensitivity analysis using the method of changing the effect model showed no significant change in the effect size after the combination, indicating that the results of this meta-analysis were stable, as shown in Table 2.

**Table 2:** Sensitivity analysis of the influencing factors of organizational citizenship behavior.

Influencing factors	Fixed effects model		Random effects model	
	SMD [95%CI]	*P* value	SMD [95%CI]	*P* value
Marital status	−0.27 [−0.33, −0.21]	<.00001	−0.27 [−0.40, −0.14]	<.0001
Employment types	−0.27 [−0.36, −0.18]	<.00001	−0.25 [−0.39, −0.10]	.0008
Professional titles	−0.38 [−0.46, −0.29]	<.00001	−0.37 [−0.52, −0.22]	<.00001
Years of work experience	−0.44 [−0.50, −0.38]	<.00001	−0.44 [−0.55, −0.33]	<.00001
Education level	−0.07 [−0.14, 0.01]	.08	−0.07 [−0.14, 0.01]	.08
Participation in mental health-related training	−0.29 [−0.40, −0.18]	<.0001	−0.29 [−0.40, −0.18]	<.0001

CI = confidence interval, SMD = standardized mean difference.

### 3.6. Publication bias

Egger’s test was used to analyze publication bias when the number of studies included in the primary outcome index was ten or more. Egger’s test for marital status (*P* = .977) and Egger’s test for years of work experience (*P* = .909) showed no publication bias.

## 4. Discussion

### 4.1. Organizational citizenship behavior

OCB indicates employees’ voluntary behaviors, which increase performance and productivity.^[28]^ To a certain extent, nursing quality depends on the organizational citizenship behavior of nursing staff.^[30]^ The quality of nursing work is closely related to a patient’s quality of life.^[31]^ Therefore, it is essential to clarify the current status of nurses’ organizational citizenship behavior. Twenty articles reported that the level of nurses’ organizational citizenship behavior was at medium,^[11,16,26]^ upper-middle^[12–15,20–23,29]^ and high.^[10,17–19,24,25,27,28]^ It can be seen that most of the literature reports that nurses have a relatively high level of organizational citizenship behavior. Zeng et al^[29]^ believed nurses’ organizational citizenship behavior was at an upper-middle level during the COVID-19 pandemic. Studies have shown that creating a favorable ethical climate can improve nurses’ organizational citizenship behavior.^[10,24,25]^

### 4.2. The influencing factors of organizational citizenship behavior

Research has shown that married nurses have higher organizational citizenship behaviors than unmarried nurses. This conclusion is consistent with the results of Leng YN et al,^[10]^ Lu et al,^[20]^ and Sha^[23]^ believed that married nurses’ profound family responsibility and caring behavior could naturally extend to patient care and effectively stimulate their organizational citizenship behavior. Family emotional communication is often the best way to provide psychological support and relieve work stress.^[32]^ Due to the lack of family support brought by marriage, unmarried nurses find it difficult to relieve their work pressure and reduce their work enthusiasm.^[29]^ Nursing managers should pay more attention to unmarried nurses’ physical and mental state and provide more organizational care and emotional support.

The meta-analysis showed that state-employed nurses’ organizational citizenship behavior was higher than that of contract-employed nurses. This conclusion is consistent with the results of Lu et al.^[20]^ Compared with state-employed nurses, contract-employed nurses have differences in terms of contract terms, salary and benefits, promotion opportunities, career development, etc,^[25]^ which makes contract-employed nurses have a poor sense of professional belonging.^[33]^ Therefore, nursing managers should optimize contract nurses’ treatment and career development to enhance their sense of belonging and organizational citizenship behavior.

The influence of educational level on nurses’ organizational citizenship behavior is still controversial. The meta-analysis of this study showed no statistically significant difference in the scores of organizational citizenship behavior of nurses with different education levels (*P* = .08). However, Sha^[23]^ and Wang et al^[25]^ pointed out that educational background impacted nurses’ organizational citizenship behavior. This may be related to regional cultural differences and the diversity of education systems.

The meta-analysis of this study showed that the score of organizational citizenship behavior of nurses who participated in mental health-related training was higher than that of nurses who did not participate in mental health-related training. The difference was statistically significant (*P* < .00001). Jin et al^[27]^ and Zeng et al^[29]^ found that psychological capital is a protective factor for OCB. Studies have shown that mental health-related training can be used effectively for nurses to tap into and develop psychological resources.^[19,34]^ A South African academic has suggested that mental health-related training be integrated into vocational training programs for nurses.^[26]^ Some researchers believe that nurses taking psychology courses at school have more positive psychological resources.^[35]^ Therefore, medical institutions should consider developing mental health-related training courses to stimulate nurses’ organizational citizenship behavior.

The meta-analysis of this study showed that nurses with nurse-in-charge or above titles had higher organizational citizenship behavior scores than those with nurse practitioner or below titles (*P* < .0001). This conclusion is consistent with the results of Yu et al.^[18]^ Nurses with high professional titles have good social networks, which can more effectively promote citizenship behavior within the organization.^[13]^ The study found that nurses with higher professional titles often have better emotional intelligence,^[36]^ likely to be an important driving force for them to show more organizational citizenship behavior.^[14]^ Nursing managers should encourage nurses with high titles to play a leading role, build a cross-title communication platform, and accelerate the growth process of nurses with low titles.

This meta-analysis showed that nurses with more than 10 years of working experience had high organizational citizenship behavior. This conclusion is consistent with the results of Huang and Xie.^[12]^ The reason may be that nurses with longer working years understand the organization’s development goals, and they know how to achieve the organization’s goals through their own behavior.^[37]^ Nursing managers encourage senior nurses to lead junior nurses in growing and developing together to achieve the development goals of the whole nursing team.

### 4.3. Limitations of this review

This review had several limitations. Firstly, as 95% of studies were included from Asia, that might limit the generalization of the findings of this meta-analysis. Additionally, this review only involved cross-sectional studies, and the association between variables only analyzed cannot infer causality. Finally, the regional differences in the included studies and the inconsistent measurement tools of the outcome indicators may have led to the heterogeneity among the studies.

## 5. Conclusion

In conclusion, the level of organizational citizenship behavior of clinical nurses is at a relatively high level. Marital status, employment types, participation in mental health-related training, professional titles, and years of work experience have an impact on the organizational citizenship behavior of clinical nurses. Managers can effectively encourage nurses to show organizational citizenship behavior by developing career plans, establishing good social networks and organizing regular mental health training.

Research on the impact of different healthcare settings on nurses’ OCB needs to be strengthened in the future. This research will be valuable and contribute to the field’s ongoing development.

## Acknowledgments

I would like to thank my supervisor for all the work he has done in guiding my paper.

## Author contributions

**Conceptualization:** Jia-Xi Sun, Fu-Yan Liu, Wen-Nv Hao.

**Data curation:** Jia-Xi Sun, Fu-Yan Liu, Wen-Nv Hao.

**Formal analysis:** Jia-Xi Sun, Fu-Yan Liu, Wen-Nv Hao.

**Methodology:** Jia-Xi Sun, Fu-Yan Liu, Wen-Nv Hao.

**Project administration:** Wen-Nv Hao.

**Software:** Jia-Xi Sun, Fu-Yan Liu, Wen-Nv Hao.

**Supervision:** Wen-Nv Hao.

**Validation:** Wen-Nv Hao.

**Writing – original draft:** Jia-Xi Sun.

**Writing – review & editing:** Jia-Xi Sun, Fu-Yan Liu, Wen-Nv Hao.
